# An overview of ethylene insensitive tomato mutants: Advantages and disadvantages for postharvest fruit shelf-life and future perspective

**DOI:** 10.3389/fpls.2023.1079052

**Published:** 2023-01-27

**Authors:** Syariful Mubarok, Muhammad Abdilah Hasan Qonit, Bayu Pradana Nur Rahmat, Rahmat Budiarto, Erni Suminar, Anne Nuraini

**Affiliations:** ^1^ Department of Agronomy, Faculty of Agriculture, Universitas Padjadjaran, Sumedang, Indonesia; ^2^ Master Program of Agro-Industry Technology, Faculty of Agro-Industrial Technology, Universitas Padjadjaran, Sumedang, Indonesia; ^3^ Master Program of Agronomy, Faculty of Agriculture, Universitas Padjadjaran, Sumedang, Indonesia

**Keywords:** ethylene, mutant, postharvest, tomato, receptor

## Abstract

The presence of ethylene during postharvest handling of tomatoes can be the main problem in maintaining fruit shelf-life by accelerating the ripening process and causing several quality changes in fruit. Several researchers have studied the methods for improving the postharvest life of tomato fruit by controlling ethylene response, such as by mutation. New ethylene receptor mutants have been identified, namely *Sletr1-1*, *Sletr1-2*, *Nr* (*Never ripe*), *Sletr4-1*, and *Sletr5-1*. This review identifies the favorable and undesirable effects of several ethylene receptor mutants. Also, the impact of those mutations on the metabolite alteration of tomatoes and the future perspectives of those ethylene receptor mutants. The review data is taken from the primary data of our experiment related to ethylene receptor mutants and the secondary data from numerous publications in Google Scholar and other sources pertaining to ethylene physiology. This review concluded that mutation in the *SlETR1* gene was more effective than mutation in *NR, SLETR4, and SLETR5* genes in generating a new ethylene mutant. *Sletr1-2* mutant is a potential ethylene receptor mutant for developing new tomato cultivars with prolonged fruit-shelf life without any undesirable effect. Therefore, that has many challenges to using the *Sletr1-2* mutant for future purposes in breeding programs.

## 1 Introduction

Tomato (*Solanum lycopersicum*) is a popular horticulture crop consumed as fresh fruit or raw material for the food industry. Tomato production has increased worldwide every year. Tomato contains high micro and macronutrients such as vitamins, minerals, fiber, and other beneficial compounds for human health. Furthermore, it is a model for studying fruit biology, fruit development, softening, ripening, and fruit metabolism (Brummell and Harpster, 2001; Giovannoni, 2004; Carrari and Fernie, 2006), because it has a small genome size (950 Mb), a relatively short life cycle, and stable genetic transformation (Osorio et al., 2011; Kumar et al., 2012). Tomato belongs to climacteric fruit. Thus postharvest handling is essential during shipment and marketing. In climacteric fruits, ethylene accelerates fruit ripening and softening. Moreover, ethylene affects leaf abscission, stem or root elongation, root hair development, epinasty, and flower fading (Abeles et al., 1992).

In developing countries, the loss of horticultural products during postharvest handling reached 50% due to storage, transportation, and packaging conditions (Kitinoja and Kader, 2015). Moreover, the presence of ethylene directly affects the lost fruit quality. Several methods have been developed to prevent the ethylene effect in reducing postharvest tomato fruit quality, such as inhibiting ethylene biosynthesis and perception by chemical compounds, atmosphere modification, and genetic modification. In climacteric fruits such as tomatoes, the inhibition of ethylene perception is more effective than ethylene biosynthesis due to the limitation in the perception of ethylene to its receptor. 1-Methylcyclopropene (1-MCP) is a non-toxic chemical compound that effectively prevents the binding process of ethylene to the receptor. Therefore, the ethylene effect can be minimized. However, this method can be more laborious and impracticable to apply to the farmers. Recently, the genetic modification approach has been widely used to develop prolonged fruit shelf life by down-regulated the ethylene biosynthesis and perception gene. However, this method needs to be supported and acceptable in some countries. The mutation method would be a practical approach for generating new ethylene-insensitive cultivars. Mutation in the ethylene receptor gene has successfully generated several insensitive tomato mutants, such as *Sletr1-1*, *Sletr1-2*, and *Sletr4-1* (Okabe et al., 2011; Mubarok et al., 2015; Mubarok et al., 2019). This review discusses the commercial use of the ethylene-insensitive mutants, *Nr*, *Sletr1-1*, *Sletr1-2*, and *Sletr4-1*, as potential breeding material to generate new prolonged shelf life for cultivated tomatoes. It also highlights the prospect and problems associated with using the mutants.

## 2 Ethylene biosynthesis and signaling

Fruit ripening is regulated by ethylene. Ethylene biosynthesis and signaling are modulated during the development of plant tissue and are responsible for inducing many biochemical processes (Abeles et al., 1992). Ethylene biosynthesis is subject to both positive and negative feedback regulation (Kende, 1993). Ethylene biosynthesis in higher plants has been well-characterized. 1-aminocyclopropane-1-carboxylic acid (ACC) synthase (ACS) and ACC oxidase (ACO) are enzymes of ethylene biosynthesis that have been recognized as the rate-limiting step (Yang and Hoffman, 1984; Kende, 1993). ACS activity is the critical step in controlling ethylene production, whereas ACO activity is constitutive (Yang and Hoffman, 1984; Theologis et al., 1993). The genes encoding ACS and ACO have been studied in more detail than other enzymes in the ethylene pathway. In higher plants, ACS and ACO are encoded by multigene families. Eight ACS genes (*LeACS1A*, *LeACS1B*, and *LeACS2-7*) (Zarembinski and Theologis, 1994; Oetiker et al., 1997; Shiu et al., 1998) and five ACO genes have been identified in tomatoes (Van-der-Hoeven et al., 2002).

The receptor is the crucial factor for ethylene action. A copper cofactor mediates the binding process of ethylene to the receptor (Rodríguez et al., 2010). The absence of copper cofactor caused less capability to bind ethylene. The binding site for copper could be replaced by any other metal, such as silver, due to a strong affinity issue. Silver is commonly used to inhibit ethylene perception by replacing the site of copper. This situation impedes conformational change that is typically found in the presence of copper cofactor in the receptor site. There were three domains classification of ethylene receptor protein based on its structure, i.e., sensor domain, kinase domain, and response regulator domain (Ciardi and Klee, 2001). Both amino-terminal ethylene-binding and the most highly conserved GAF are reported subdomains of the sensor domain (Aravind and Ponting, 1997).

In tomatoes, at least six ethylene receptor genes (*LeETR1–6*) were identified, and *LeETR3* is denoted as *NR* (Payton et al., 1996). The expression of each tomato receptor is different in temporal and spatial patterns depending on the development stage and external stimuli (Alexander and Grierson, 2002). *LeETR1* and *LeETR2* are expressed constitutively in all tissues throughout development, *NR* is up-regulated at anthesis, and both *NR* and *LeETR4* are up-regulated during ripening, senescence, abscission (Payton et al., 1996; Tieman et al., 2000), and pathogen infection (Ciardi et al., 2000). *LeETR5* is expressed in fruit, flowers, and during pathogen infection (Tieman and Klee, 1999).

The binding of ethylene to receptors causes conformational changes in a receptor or inactivates a receptor, resulting in the inactivation of a negative regulator of downstream ethylene signaling such as CTR1 (Kieber et al., 1993). Suppression of CTR1 activates ETHYLENE INSENSITIVE (EIN2) to act as an essential positive regulator of the ethylene signaling pathway (Wang et al., 2002). Genetic epinasty analysis of ethylene response mutants has shown that EIN2 acts downstream of CTR1 and positively signals upstream of EIN3 (Alonso et al., 1999; Wang et al., 2002). EIN3 is both necessary and sufficient for the activation of ethylene-responsive target genes and, in particular, for ERF1 (Solano et al., 1998). ERF1 belongs to a large family of plant-specific transcription factors referred to as ethylene response element-binding proteins (EREBPs) (Carrari and Fernie, 2006). Transcription factor ERF1 and other EREBPs can interact with the GCC box, which causes ethylene responses in plants (Yamamoto et al., 1999).

## 3 Strategy to minimize ethylene effect at receptor level

Ethylene has become a central problem in postharvest horticultural products. Several strategies are needed to manipulate the adverse ethylene effects leading to the maintenance of the postharvest quality of the horticultural product, including tomatoes. Developing new cultivars by mutation is one strategy for obtaining tomato mutants with long fruit shelf-life, such as *ripening-inhibitor* (*rin*), *colorless non-ripening* (*Cnr*), *non-ripening* (*nor*), *green-ripe* (*Gr*) and *Nr*.

Targeting induced local lesions in genomes (TILLING) is a general method to identify induced point mutations in the genomes of any organism. This method accelerates identifying the modified function of desired genes and selecting mutants rather than conventional mutation breeding. TILLING method has identified some mutants, for instance, *SleIF4E1* of tomato mutant, which showed potyvirus resistance (Piron et al., 2010), *CmACO1* of melon mutant, which produced long shelf-life fruit (Dahmani-Mardas et al., 2010), *Sletr1-1, Sletr1-2*, and *Sletr4-1*, which show in the reduction of ethylene sensitivity (Okabe et al., 2011; Mubarok et al., 2019).

The expression analysis of related genes of ethylene biosynthesis and perception has been widely investigated in tomato mutants. This analysis showed that each mutant has a different location where the mutation occurred. In the *Nr* mutant, a mutation occurred in the ethylene-binding domain of the NR ethylene receptor; therefore, ethylene cannot be perceived, and its response cannot be expressed (Lanahan et al., 1994; Wilkinson et al., 1995). In the ripening inhibitor (*rin*) mutant, the mutation occurred in the RIN transcription factor; therefore, autocatalytic ethylene production does not show, and the ethylene signal downstream cannot be transmitted (Vrebalov et al., 2002).

**Figure 1:**
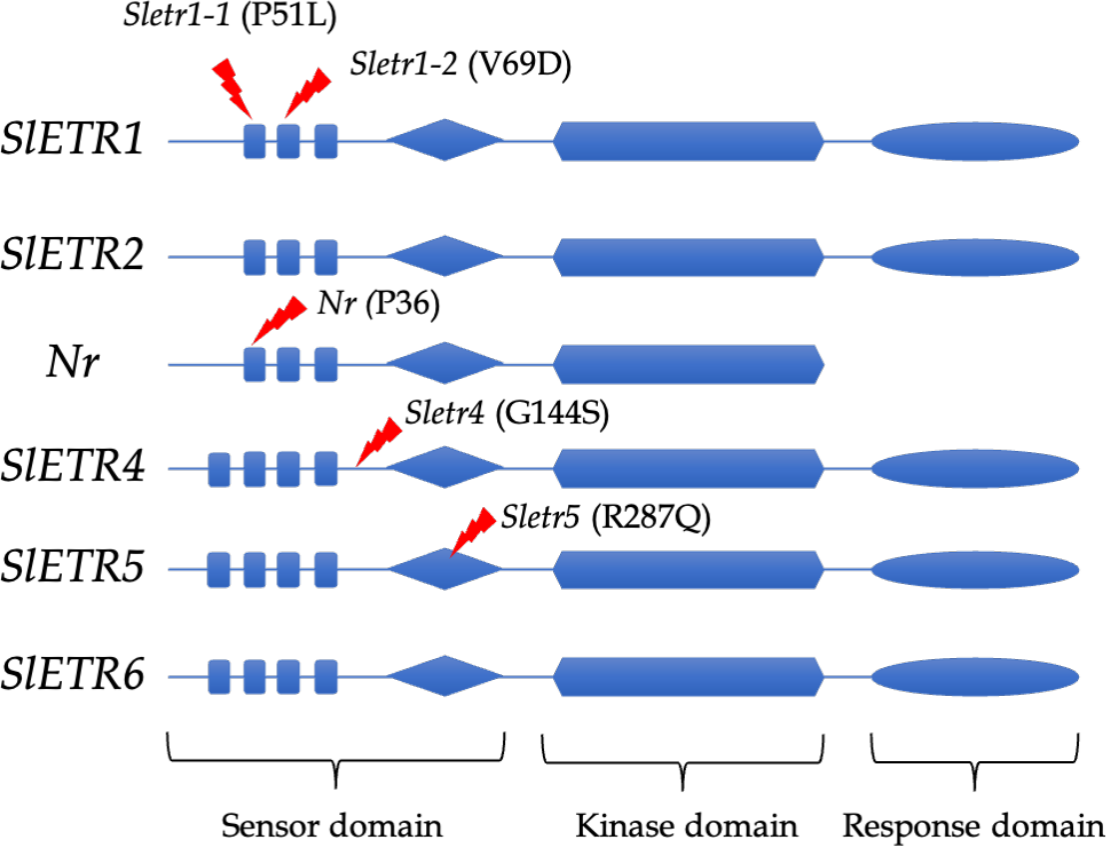
Mutation location of ethylene receptor mutants; *Sletr1-1, Sletr1-2, Nr, Sletr4-1 and Sletr5-1*.

In the novel ethylene receptor mutants, in the *Sletr1-1* and *Sletr1-2* tomato mutant of ‘Micro-Tom’, the mutations occurred in the first and second transmembrane domain in the ethylene receptor, respectively. The location of mutation of *Sletr1-1* (P51) and *Nr* (P36) are similar in the first transmembrane domain; however, they have different ethylene sensitivity (Wilkinson et al., 1995; Okabe et al., 2011). In the *Sletr4-1*, there has an amino acid substitution, G154S, that occurs between the transmembrane and GAF domains, whereas the *Sletr5-1* tomato mutant has the amino acid substitution, R278Q, within the GAF domain (Mubarok et al., 2019) (**Figure 1**).

The ethylene receptor gene plays an important role in ethylene action. Mutation in *SlETR1, SlETR4*, and *SlETR5* results in altered ethylene sensitivity, showing the different changes in ethylene triple response and fruit shelf life. From the seedlings’ ethylene triple response assay, the four new ethylene receptor mutants, *Sletr1-1, Sletr1-2, Sletr4-1*, and *Sletr5-1*, exhibited a different ethylene sensitivity. *Sletr1-1, Sletr1-2, Sletr4-1*, and *Sletr5-1* display completely ethylene insensitive, moderate ethylene insensitive, low ethylene sensitivity, and high ethylene sensitivity, respectively (Okabe et al., 2011; Mubarok et al., 2015; Mubarok et al., 2019).

## 4 Favorable effects of ethylene receptor mutant on fruit ripening

Tomato fruit development could be divided into three phases, namely (i) the main phase with rapid and active cell division; (ii) the phase with a stable increase in size due to cell expansion, and the fruit ripening phase (Pirrello et al., 2012). During the ripening process, the tomato fruit experienced specific changes in appearance, color, texture, taste, and aroma (Giovannoni, 2004). The ripening process in tomato fruits was further divided into three phases, i.e., mature green, breaker, and red. Tomato fruit discoloration during the ripening process occurred due to the increased lycopene and beta carotene content; and chlorophyll degradation during the transition from chloroplasts to chromoplasts. The mature green stage is the final fruit formation stage, as indicated by fully expanded fruit size. In this stage, seed formation began. In a later phase, the breaker, the fruit starts to rip as characterized by specific metabolites degradation and the initiation of ethylene production spike and respiration as a sign of climacteric characteristics. While in the red phase, the fruit is considered ripe, with optimum metabolite content, and also experiences the beginning of the senescence phase (Fraser et al., 1994; Osei et al., 2017).

The fruit shelf life is one of the essential characteristics of the postharvest quality of horticulture crops. In climacteric fruit such as tomatoes, fruit shelf life is commonly affected by ethylene, which accelerates fruit ripening. Therefore, to improve fruit shelf life, the response of ethylene must be minimized. Mutation in ethylene receptor genes significantly underestimated the ethylene response by extending fruit shelf life. Improving the fruit shelf life of tomatoes by developing a new cultivar is an excellent way to get a significant aim in tomato breeding because it can provide various benefits for both tomato producers and consumers. Besides long fruit shelf life, other traits must be improved in tomatoes, such as fruit performance and fruit nutrient, because it is crucial factors for fruit quality and the human diet.

Several mutant alleles, such as *Sletr1-1, Sletr1-2*, and *Nr*, reduced ethylene sensitivity, impacting fruit development and ripening. In both homozygous and heterozygous *Sletr1-1* mutants, there is a disturbance in the process of petals withering, while the effect is weaker for Nr in both homozygous and heterozygous conditions. However, this condition is influenced by parental background (Okabe et al., 2011). An earlier study by Okabe et al. (2011) reported that petal flowers of *Sletr1-1* mutants still stick to the fruit even up to 60 days after pollination (DAP), while in WT-MT and *Sletr1-2*, the petal withered at 3 and 5 DAP, respectively.

The delay in petal abortion can be used as one of the indicators related to ethylene sensitivity that further affects fruit development, ripening, and postharvest fruit shelf life. The ripening phenotypes in *Sletr1-1* were different between homozygous and heterozygous plants. The homozygous *Sletr1-1* displayed yellow and orange color (Okabe et al., 2011) whereas heterozygous *Sletr1-1* fruits showed reddish-orange color. Homozygous *Sletr1-1* and *Nr* showed similar fruit ripening phenotypes due to imperfect ripening processes (Lanahan et al., 1994; Okabe et al., 2011). Phenotypic differences in fruit ripening were not detected between homozygous and heterozygous *Sletr1-2*, where the fruit showed perfect ripening. Crossing commercial tomato cultivars with several mutants, such as *Nr, Sletr1-1*, and *Sletr1-2*, might facilitate the development of a commercial F1 hybrid line. However, not all ethylene-insensitive mutants can be used as genetic material in the breeding program of long fruit shelf-life tomatoes, for example, *Nr* and *Sletr1-1*. Although both *Nr* and *Sletr1-1* had low sensitivity to ethylene, these two mutants displayed an incomplete maturation phenotype, even though they had an insufficient red color in heterozygous form.

The fruit shelf life of the mutant differs from one another either in homozygote or heterozygous form. In homozygous conditions, the fruits of *Sletr1-1* and *Sletr1-2* are still intact. They do not show any damage characterized by the absence of a black spot on the fruit surface during the 60 days of storage in a sealed chamber at 25°C, while the WT-MT fruit shows some damage at the age of 20-25 days after harvesting (Okabe et al., 2011). However, the increase in fruit storage resistance is not very strongly shown in the homozygous *Sletr4-1* (Mubarok et al., 2019). It was likely that the fruit shelf life could be dramatically extended, and post-harvest fruit damage could be inhibited. The use of *Sletr1-2* as breeding material to form a hybrid generation has been carried out. The *Sletr1-2* has a strong inheritance pattern in increasing the shelf life of fruits in all commercial parental backgrounds. However, the length of fruit shelf-life resistance is different in each parental background of ‘Aichi First’, ‘Ailsa Craig’, ‘Moneymaker’, and ‘M82’, with an average increase in shelf-life resistance ranging from 4−5 days longer in open room conditions at a storage temperature of 20 ± 2°C and relative humidity of 80% (Mubarok et al., 2015).

## 5 Undesirable effects of ethylene receptor mutant

Mutations in the *SlETR1, SLETR4*, and *SlETR5* genes do not affect plant external appearance, especially in vegetative organs. However, there is an alteration in plant sensitivity to abiotic stress. The *Sletr1-1* is a promising genotype since it has low sensitivity to ethylene and shows dominant inheritance during a breeding program in increasing the shelf life of tomato fruits (Okabe et al., 2011). However, some undesirable characteristics are found in the mutant *Sletr1-1*, i.e., stress sensitivity response. The F1 generations of *Sletr1-1* experience withering and disease attacks during the transplanting process to the NFT system. The wilting plant is caused by root damage when transplanted from the nursery (Mubarok et al., 2015). The inability of the F1 *Sletr1-1* to recover the damaged root system and the inhibition of new root formation cause secondary threats, such as the pathogen attack to the root and stem base. In contrast, the F1 *Sletr1-2* are not susceptible to biotic and abiotic stress, whereas all hybrid of F1 *Sletr1-2* shows similar characteristics to F1 WT-MT, i.e., healthy and white roots without roots rot after transplanting (Mubarok et al., 2015).

Heterozygous *Sletr1-1* shows increased susceptibility to infections of diseases caused by *Fusarium oxysporum*. A similar finding was reported in the *Atetr1-1* mutant of *Arabidopsis thaliana* that showed an increase in disease infection by certain pathogens such as *Botrytis cinerea, Fusarium solani, Fusarium oxysporum* f. sp. matthiolae, *Xanthomonas campestris* pv. Campestris, and *Pythium* spp (O'Donnell et al., 2003; Agarwal et al., 2012). In addition, *Nr* mutants cannot produce adventitious roots in waterlogging conditions (Visser and Voesenek, 2004; Vidoz et al., 2010) and are susceptible to some pathogens (Francia et al., 2007; Kavroulakis et al., 2007; Cantu et al., 2009).

Aside from the increased susceptibility to biotic and abiotic stress, another undesirable characteristic in the ethylene mutant is a change in fruit color as the implication of the pigment reduction, especially lycopene and beta carotene. In tomatoes, the fruit color can be used to estimate maturity level (**Table 1**). The delay in fruit ripening occurs in the mutant *Sletr1-1*. This mutant *Sletr1-1* undergoes yellow or orange discoloration 7 to 10 days later than WT-MT. Moreover, this mutant mostly does not produce full red fruit color nor does the F1 generation (Okabe et al., 2011; Mubarok et al., 2015). The inability to produce full red color is also observed in the mutant *Nr* (Lanahan et al., 1994). On the opposite, this phenomenon is not found in the mutant *Sletr1-2* or its F1 generation. *Sletr1-2* fruits can produce the normal red fruit color as its wild type even in their F1 generation (Okabe et al., 2011; Mubarok et al., 2015). Although the mutants *Sletr1-1* and *Nr* produce fruits with a long shelf life, they have yet to be widely used in breeding programs due to their susceptibility to biotic and abiotic stress, and the imperfection of the fruit ripening process leads to less red color (Francia et al., 2007; Kavroulakis et al., 2007; Cantu et al., 2009)

**Table 1:** The differences between four ethylene receptor mutants of tomato.

No.	Characteristics	*Sletr1-1 (* Okabe et al., 2011; Mubarok et al., 2015 *)*	*Sletr1-2 (* Okabe et al., 2011; Mubarok et al., 2015 *)*	*Sletr4-1 (* Mubarok et al., 2019 *)*	*Sletr5-1 (* Mubarok et al., 2019 *)*
1	Amino Acid Substitution	P51L	V69D	G154S	R278Q
2	Mutation location	The first transmembrane domain of *SlETR1*	The second transmembrane domain of *SlETR1*	Between the transmembrane and GAF domains of *SlETR4*	Within the GAF domain of *SlETR5*
3	Ethylene sensitivity	Completely ethylene insensitive	Moderate ethylene insensitive	Low ethylene insensitive	Increased ethylene sensitivity
4	Plant appearance	Not change	Not change	Not change	Not change
5	Leaf shape	Not change	Not change	Not change	Not change
6	Fruit color	Yellow to Orange	Red Light	Red	Red
7	Fruit size	Not change	Not change	Not change	Not change
8	Fruit firmness	Harder	Harder	Not change	Not change

## 6 Metabolite alteration of ethylene-insensitive mutants

The change or mutation in related genes in ethylene biosynthesis and action may regulate the gene transcription and ultimately affect the metabolite contents of the tomato fruit. In the *rin* mutant, the mutation in the *RIN* gene inhibits carotenoid biosynthesis, aroma, production of flavor compounds, and softening (Herner and Sink, 1973; Tigchelaar et al., 1978; Knapp et al., 1989; Vrebalov et al., 2002; Kumar et al., 2012). The novel insights into the molecular biology of ethylene-mediated ripening regulatory networks in tomato during fruit development has been revealed by analyzing *nor, rin*, and *Nr* mutant at transcriptomic, proteomic, and metabolomic levels (Osorio et al., 2011). Recently, a new investigation on the effect of the mutation in *rin* mutant showed that the *RIN* mutation results in a profound change in fruit transcriptome during ripening that is similar to other spontaneous mutations, such as *Nr, hp-2dg*, and *cnr* (Eriksson et al., 2004; Alba et al., 2005; Osorio et al., 2011; Rohrmann et al., 2011; Kumar et al., 2012; Mubarok et al., 2021).

### 6.1 Sugar

Ethylene accelerates fruit ripening, which contributes to changes in the nutrient content; however, it also accelerates quality deterioration by shortening the shelf life of the fruit. There is a change in the total sugar content during fruit maturation that can be used to determine the sweetness of tomato fruits (Mubarok et al., 2019). The entire sugar content of the mutant *Sletr1-1* and *Sletr1-2*, both in homozygous and heterozygous form, is lower than WT-MT, except for the F1 *Sletr1-2* heterozygous (Mubarok et al., 2016). The fruit of nor mutant has the lowest total sugar content, followed by the fruit of *Nr, rin, Sletr1-1*, and *Sletr1-2* (Osorio et al., 2011; Mubarok et al., 2016; Mubarok et al., 2019; Osorio et al., 2020). The difference in the sensitivity to ethylene can cause the variation in sugar content in these mutants. Mutant tomato plants, namely *nor, Nr*, and *rin*, have complete ethylene insensitivity character (Osorio et al., 2011), while the mutant *Sletr1-1* and *Sletr1-2* have partial ethylene insensitivity character. The variation of ethylene sensitivity may affect the expression of genes that regulate the conversion of starch into sugars (Osorio et al., 2020), thus leading to the difference in sugar content. Baldwin et al. (1998) stated that glucose and fructose are the tomato’s main sugar components contributing to the sweetness level. Under the heterozygous line of the F1 *Sletr1-2*, the *Sletr1-2* mutation did not significantly affect the changes of sucrose, fructose, and glucose under different pure-line cultivar parents. These results contrast with previous studies in the homozygous line of *Nr*, *nor*, and *rin*, demonstrating the reduction in sucrose, glucose, and fructose levels (Hobson, 1980; Osorio et al., 2011).

### 6.2 Total soluble solid

Sugar content is preliminarily studied as the total soluble solids (TSS) variable, including in the tomato study (Mubarok et al., 2019; Mubarok et al., 2021). The TSS in tomato fruits increased in line with the ripening process (Mubarok et al., 2019), as the impact of the conversion of starch into sugar and the hydrolysis of polysaccharide cell walls to hemicellulose and pectin during the maturation process (Mubarok et al., 2022). In general, the stronger the red color observes, the higher the ripening level of the tomato fruit and the higher the TSS content. Before fully ripe, the starch content in the fruit can reach 20% of the dry weight; then it is degraded into other compounds, such as sugar (Ho, 1996).

Mutant tomatoes, namely *Sletr1-1, Sletr1-2, Sletr4-1, Sletr5-1, rin*, and *nor*, have lower TSS content than WT-MT tomatoes on all maturity stadia (Mubarok et al., 2015; Mubarok et al., 2019; Mubarok et al., 2019). The *rin* and *nor* mutant have similar TSS, i.e., 4.6°Brix, and this result is still lower than the TSS value of *Sletr1-1*, *Sletr1-2, Sletr4-1*, and *Sletr5-1 (* Mubarok et al., 2015; Mubarok et al., 2019; Mubarok et al., 2019 *).* This phenomenon is associated with the lower expression of genes that regulate the activity of pectinase in *rin* and nor tomatoes (Osorio et al., 2020), compared to *Sletr1-1, Sletr1-2, Sletr4-1*, and *Sletr5-1*. So, the amount of pectin converted by pectinase during storage is also lower (Osorio et al., 2011).

### 6.3 pH and titratable acidity

Aside from the sugar content indicated by the TSS variable, the alteration in the mutant is also found in terms of acidity level. Fruit pH and titratable acidity (TA) are two common variables used to determine the acidity level of tomato fruit (Mubarok et al., 2019). Along with TSS, acidity variables form the balance of sour and sweet in the fruit taste profile and post-harvest quality in tomato fruits (Grierson and Fray, 1994). An earlier study by Tran et al. (2017) showed that the total acid content increase during fruit formation and enlargement. However, it declines in line with the ripening process due to the degradation of organic acids during ethylene biosynthesis in the respiration stage.

The fruit TA value of insensitive ethylene mutants, namely *rin, nor, Sletr1-1, Sletr1-2*, *Sletr4-1*, and *Sletr5-1*, are higher than WT-MT. Lobit et al. reported that the TA and fruit pH are closely related (Lobit et al., 2002). The increase in TA is accompanied by a decrease in the pH. Therefore, the pH of the insensitive ethylene mutant of *rin, nor, Sletr1-1, Sletr1-2, Sletr4-1*, and *Sletr5-1* is lower than WT-MT (Mubarok et al., 2015; Mubarok et al., 2019; Mubarok et al., 2019).

### 6.4 Lycopene, beta carotene

In addition to TSS and TA, some phytochemicals, such as lycopene, beta carotene, flavonoid, and polyphenols, are reported to differ in ethylene insensitive mutant compared to its wild type, leading to the variation of antioxidant activity. Lycopene is a carotenoid responsible for reddish color formation on tomato fruits (Rai et al., 2013). The *rin* and nor have the lowest lycopene content. This finding can be caused by the low expression of genes that play a role in the process of lycopene formation, namely *PSY1, PSY2, PDS, ZDS*, and *CRTISO* genes (Kitagawa et al., 2005; Osorio et al., 2020). The *Sletr1-1* and *Sletr1-2* have a higher lycopene content than *rin* and *nor*, but they are still lower than the WT-MT (Mubarok et al., 2015; Mubarok et al., 2019; Mubarok et al., 2019). The findings show that ethylene may associate with the formation of lycopene.

Beta carotene in ethylene insensitive mutant is lower than that in WT-MT. The *rin* and *nor* have the lowest beta carotene content. It may be caused by the low activity of the *CRTR-b*1 gene, which converts γ-carotene into beta-carotene (Osorio et al., 2020). The mutant *Sletr1-1* and *Sletr1-2* have higher beta carotene than the *rin* and *nor* mutant *(* Mubarok et al., 2015; Mubarok et al., 2019 *).* This phenomenon can be associated with the difference in ethylene insensitivity levels between mutant genotypes.

### 6.5 Polyphenols and flavonoids

The content of polyphenols and flavonoids in the fruit of ethylene-insensitive mutant tomatoes varies in response to genotypic factors. The content of polyphenols and flavonoids in *rin* and *nor* mutant is very low (Minoggio et al., 2003). Meanwhile, the polyphenol content in fruits of the *Sletr1-1* and *Sletr1-2* is not significantly different from WT-MT, whereas the flavonoid content in these mutants is lower than in WT-MT (Mubarok et al., 2019). The rate of polyphenol and flavonoid content, from low to high, can be sorted as follows; *rin, nor, Sletr1-1, Sletr1-2*, and WT-MT. The lower polyphenols and flavonoids in ethylene-insensitive mutants have ascertained ethylene’s involvement in the biosynthesis of polyphenols and flavonoids (Chaudhary et al., 2018). Meanwhile, the difference in flavonoid and polyphenol content among mutants can be caused by differences in the degree of insensitivity level to ethylene (Osorio et al., 2020).

### 6.6 Antioxidant activity

The antioxidant activity of ethylene insensitive mutant, namely *rin*, is the lowest, followed by *nor (* Minoggio et al., 2003 *)*, *Sletr1-1*, and *Sletr1-2.* The antioxidant activity of *Sletr1-2* is not significantly different from WT-MT (Mubarok et al., 2019), while the antioxidant activity of *rin, nor*, and *Sletr1-1* is lower than WT-MT. This situation is associated with the lower content of lycopene, beta carotene, flavonoids, and polyphenols on *rin, nor*, and *Sletr1-1* rather than *Sletr1-2* and the WT-MT (Minoggio et al., 2003; Kitagawa et al., 2005; Mubarok et al., 2019; Osorio et al., 2020). Earlier study reported that both lycopene and beta carotene are potent antioxidant compounds whose content dramatically affects the rate of antioxidant activity of tomato fruit (Mubarok et al., 2019).

### 6.7 Organic acids

The presence of organic acids correlates with fruit quality that directly affects fruit sourness, such as tomato. Tang et al. (2010) stated that the organic acid content is essential in food nutrition. The primary organic acids in tomato fruit, namely citrate, and malate (Baldwin et al., 1998). Oms-Oliu et al. (2011) stated that the metabolisms of citrate and malate are subjected to ethylene regulation. Several factors affect the levels of organic acids in tomato fruit, and ethylene is one of the influencing factors (Mubarok et al., 2016). The change of organic acid content in fruit is directly affected by the function of ethylene response. Inhibition of the ethylene perception due to a mutation in the ethylene receptor gene significantly increased the total organic acid content (Osorio et al., 2011; Mubarok et al., 2016). Mubarok et al. (2016) stated that the F1 generation of *Sletr1-1* and *Sletr1-2* mutants have a higher total organic acid, malate, and citrate content than the control. High organic acid content was also detected in the *Nr* mutant due to a mutation in the ethylene receptor gene (Osorio et al., 2011).

### 6.8 Amino acids

The ethylene was not directly affecting the change in fruit amino acids. Mubarok et al. (2016) reported that the variation of the amino acids in four F1 generations of *Sletr1-2* was dependent on the genetic background. Although the *Sletr1-2* mutation did not directly affect the total amino acids, it significantly induced changes in the individual amino acid levels, such as glutamic acid, glutamine, aspartic acid, and GABA (Mubarok et al., 2016). Oms-Oliu et al., 2011 stated that those four amino acids are the primary amino acids in the tomato fruit (Oms-Oliu et al., 2011). Different behaviors in accumulating individual amino acids were also observed in the *Nr* mutant^.56^ Regarding fruit taste quality, glutamic acid substantially enhances taste perception or fruitiness intensity that correlates with fruit shelf life (Yilmaz, 2001; Oms-Oliu et al., 2011). Associations between long fruit shelf life and lower levels of glutamic acid have been demonstrated in the *Nr* mutants. Still, it was not shown in the *Sletr1-2* F1 (Pratta et al., 2004; Osorio et al., 2011). The *Sletr1-2* F1 hybrid showed no change in the level of glutamic acid compared with the WT-MT F1 hybrid line fruit. Based on this study, we conclude that *Sletr1-2* F1 can produce red fruit and glutamic acid that did not influence the postharvest fruit quality (Oms-Oliu et al., 2011).

## 7 Future perspective

The presence of ethylene hormone can affect the growth, yield, and quality of horticultural commodity yields. In post-harvest handling, the presence of ethylene can have both positive and negative effects depending upon the purpose of its use. For storage and transportation purposes, especially in climacteric fruits such as tomatoes, ethylene accelerates the fruit ripening, leading to shorter fruit shelf life. Mutant with ethylene gene receptor modification can be used as an alternative solution because the negative influence of the ethylene hormone in this genotype can be minimized.

These mutants can be used as elders in plant breeding programs to produce new superior tomato cultivars with longer fruit shelf life (Mubarok et al., 2015; Wiguna et al., 2021). With the production of this shelf-resistant commercial tomato cultivar, the post-harvest problem in tomato fruits can be solved. The use of these mutant tomatoes for breeding programs will be more effective when compared to other ethylene-inhibition methods, such as controlled atmospheric storage with high-cost disadvantages. In the future, these mutants will have a considerable function, especially in plant breeding programs to assemble tomatoes for fresh consumption. With the knowledge of these mutants, it is hoped that the breeding program can run well and that new superior tomato cultivars can be produced, especially for the raw consumed tomatoes such as beef and cherry tomatoes.

## 8 Conclusions

Ethylene is one of the critical problems in the post-harvest handling of climacteric fruits such as tomatoes. Developing tomato cultivars that are insensitive to ethylene is one of the effective ways to control the negative influence of ethylene in accelerating fruit damage. The TILLING method has successfully obtained mutant tomatoes less sensitive to ethylene, including *Nr*, *Sletr1-1, Sletr1-2*, and *Sletr4-1*. The *Sletr1-2* mutant is the most promising genotype for further development among the four mentioned mutant genotypes. The *Sletr1-2* mutant is less sensitive to environmental stress and can produce red fruits, unlike the *Sletr1-1* mutant, which only has yellow fruits. In its F1 generation, the mutation in the *Sletr1-2* allele shows a less significant effect on the nutritional content of the fruit, which is very important for human health. Therefore, the *Sletr1-2* mutant is a potential mutant used in breeding programs for assembling new superior tomato cultivars with long fruit shelf life.

## Author contributions

All authors contributed to the article and approved the submitted version.
